# Scoliosis as a Paradigm of Pathological Spinal Curvature: Molecular Mechanisms and Imaging Innovations: Molecular and Imaging Insights in Scoliosis

**DOI:** 10.31661/gmj.v14i.3814

**Published:** 2025-11-05

**Authors:** Alireza Ghanbari, Tohid Emami Meybodi, Bahare Nezhadmohammad Namaghi, Tohid Khalili Bisafar, Majid Jahanshahi, Karo Khosravi, Khatere Mokhtari, Babak Roshanravan

**Affiliations:** ^1^ Bone and Joint Reconstruction Research Center, Department of Orthopedic, School of Medicine, Iran University of Medical Sciences, Tehran, Iran; ^2^ Neuroscience Research Center, Iran University of Medical Sciences, Tehran, Iran; ^3^ Functional Neurosurgery Research Center, Shohada Tajrish Hospital, Shahid Beheshti University of Medical Sciences, Tehran, Iran; ^4^ Student Research Committee, School of Medicine, Shafa Orthopedic Hospital, Iran University of Medical Sciences, Tehran, Iran; ^5^ Department of Neurosurgery, School of Medicine, Iran University of Medical Sciences, Tehran, Iran; ^6^ Department of Cell and Molecular Biology and Microbiology, Faculty of Biological Science and Technology, University of Isfahan, Isfahan, Iran; ^7^ Department of Orthopedic Surgery, School of Medicine, Imam Reza Hospital, Birjand University of Medical Sciences, Birjand, Iran

**Keywords:** Spinal Curvature, Scoliosis, Imaging Techniques, Spinal Deformities, Molecular Mechanisms

## Abstract

Pathological spinal curvature encompasses a broad spectrum of deformities that arise from a complex interplay of genetic, molecular, and biomechanical factors. This review synthesizes current knowledge on the molecular underpinnings of spinal deformities, with a focus on the dysregulation of non-coding RNAs, aberrant activation of the Wnt signaling pathway, inflammatory cytokine imbalances, and epigenetic modifications. In parallel, the article provides a detailed overview of both conventional and emerging imaging techniques used in the clinical assessment of spinal curvature. Traditional radiographic methods, such as Cobb angle measurement and Ferguson’s method, are critically compared with advanced modalities—including surface topography, ultrasound imaging, and computer-aided 3D reconstructions—that promise enhanced diagnostic accuracy while minimizing radiation exposure. By bridging molecular insights with clinical imaging advancements, this review underscores the importance of an integrated diagnostic approach for early detection and effective management of scoliosis and related spinal deformities. The convergence of these disciplines not only enriches our understanding of the pathogenesis of spinal curvature but also lays the foundation for the development of personalized therapeutic strategies.

## Introduction

Humans depend on their spines for different tasks, including bearing their weight. These tasks are interrupted if the spine is not in a normal state. A normal spine is centered on the pelvis with two normal lumbar and thoracic curves [1]. The presence of any other curves in the coronal, sagittal, or axial planes categorizes the spine as abnormal [2]. The different spinal deformities are de-novo scoliosis, adolescent idiopathic scoliosis, hyperkyphosis, iatrogenic sagittal deformity, focal deformity due to multiple degenerative disc disease with global deformity, and post-traumatic spinal deformity. The known etiologies for spinal deformities include de-novo, degeneration, and trauma [3].

Scoliosis is defined as an abnormal lateral curvature of the spine—operationally determined by a Cobb angle greater than 10 degrees—and is consistently accompanied by various levels of hyperlordosis and rotational deformities [4][5][6]. Scoliosis encompasses several subtypes—idiopathic, syndromic, neuromuscular, and congenital—with adolescent idiopathic scoliosis (AIS) being the most common, affecting approximately 1%-4% of adolescents globally [7][8]. Another common cause of scoliosis is congenital spinal malformation, which arises during embryogenesis and results in mixed segmental vertebral deformities [9]. The etiology of scoliosis is multifactorial, involving an interplay of both environmental and genetic factors. For example, environmental factors such as maternal alcohol consumption and vitamin deficiencies during pregnancy are implicated in the development of congenital scoliosis. Additionally, genetic variations, including single-nucleotide polymorphisms (SNPs) in genes like LBX1, GPR126, BNC2, and PAX1, have been associated with idiopathic scoliosis [10][11][12][13][14][15][16][17][18][19]. However, the precise cellular and molecular mechanisms connecting these etiological factors to scoliosis development remain largely unclear. Therefore, investigating the molecular pathogenesis of scoliosis is essential for identifying novel molecular markers that enable early detection of at-risk individuals and for advancing mechanism-driven therapeutic strategies.

The population is growing older in different areas, including the United States. Given the higher prevalence among this group of individuals, this old population is expected to be challenged with spinal deformities [20]. Given the rising costs associated with managing spinal deformities, early diagnosis and effective treatment strategies are becoming increasingly important. This narrative review explores the molecular and cellular mechanisms underlying scoliosis, with a particular focus on the role of non-coding RNAs and other molecular regulators. Additionally, it summarizes the currently available imaging techniques for assessing pathological spinal curvatures, aiming to bridge the gap between molecular insights and clinical applications.

## Non-coding RNAs in Scoliosis

Non-coding RNAs (ncRNAs) represent a crucial class of regulatory transcripts that do not encode proteins. They are broadly classified into three major subclasses: long non-coding RNAs (lncRNAs), microRNAs (miRNAs), and circular RNAs (circRNAs) [21][22][23][24]. While the mechanism by which miRNAs regulate gene expression is relatively straightforward—guiding the RNA-induced silencing complex (RISC) to target mRNAs through base-pairing, leading to their degradation and/or translational inhibition—lncRNAs and circRNAs exert regulatory effects at multiple levels. LncRNAs can modulate gene expression through mechanisms such as DNA methylation, histone modification, recruitment of transcription factors, miRNA sponging, and regulation of mRNA stability. In contrast, circRNAs influence gene expression by acting as miRNA sponges, regulating transcription, modulating alternative splicing, directly interacting with RNA-binding proteins, and facilitating protein translation through rolling circle amplification [25][26][27]. ncRNAs serve as pivotal regulators that coordinate essential cellular processes, including cell proliferation, programmed cell death, autophagy, differentiation, metabolism, migration, and invasion [28][29][30][31][32][33]. Consequently, it is not surprising that ncRNAs are frequently dysregulated across a wide array of diseases, including neoplastic, inflammatory, and metabolic disorders [34][35][36][37][38][39]. From a clinical perspective, the frequent changes observed in ncRNA levels in body fluids such as saliva, blood, and urine during disease states highlight their potential as promising biomarkers for early diagnosis and prognosis [40][41][42].

A growing body of evidence indicates that abnormal ncRNA expression plays a pivotal role in the development of orthopedic disorders, including osteosarcoma, osteoporosis, osteoarthritis, and intervertebral disc degeneration [29][32][43][44][45]. Emerging evidence also suggests that ncRNAs are dysregulated in scoliosis and contribute functionally to its pathogenesis [39][46][47].

Non-coding RNAs (ncRNAs) not only serve as biomarkers but also actively contribute to scoliosis pathogenesis by modulating key developmental signaling pathways. Several studies have shown that dysregulated miRNAs in scoliosis—such as miR-122-5p and miR-223-5p—can directly target components of the TGF-β signaling cascade, which is known to regulate extracellular matrix remodeling and chondrogenesis during spinal development [48][49][50][51]. For instance, downregulation of miR-1306-3p may relieve repression on SMAD family genes, thereby amplifying TGF-β signaling and altering growth plate organization. Similarly, lncRNAs such as ENST00000440778.1 have been implicated in the regulation of osteogenic transcription factors (e.g., Runx2) and may act as competitive endogenous RNAs (ceRNAs), sponging miRNAs like miR-27a-5p that target genes within the Hedgehog and Notch pathways. These pathways are essential for proper segmentation, vertebral ossification, and musculoskeletal coordination. Furthermore, circRNAs, through their interactions with RNA-binding proteins and modulation of mRNA stability, influence not only the mechanical properties of the spine but also cellular polarity and proliferation within the vertebral growth plates. By dissecting these mechanistic links, future research may reveal ncRNA-based therapeutic strategies to prevent or slow the progression of scoliosis [52][53][54][55][56][57].

Profiling ncRNA expression through whole-transcriptome sequencing, microarray, or PCR array, followed by validation using reverse transcription (RT)-quantitative PCR, is the most widely used approach for identifying and confirming dysregulated ncRNAs in specific disease conditions [21][39][58][59].

**Figure-1:**
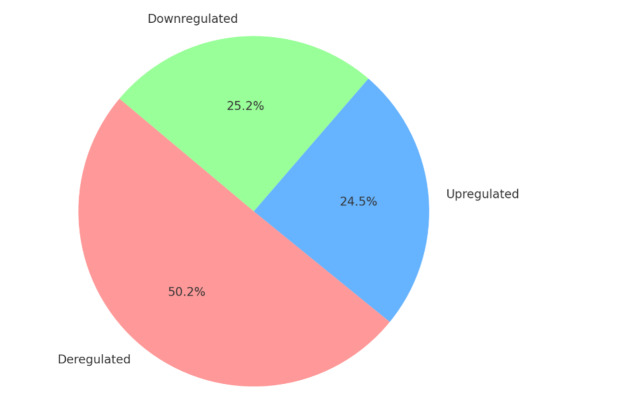
Figure 1. ncRNAs expression profiles in scoliosis

**Figure-2:**
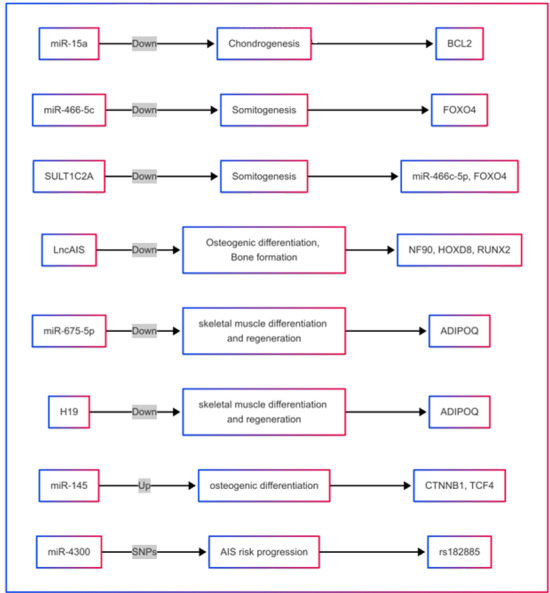
Figure 2. Functionally characterized ncRNAs in scoliosis ([47, 52, 61-64]).

Several studies have utilized various molecular techniques to explore gene and microRNA expression in different clinical conditions, employing stringent filtering criteria to identify deregulated molecules. One study focused on AIS cases compared to healthy children, using microarray and RT-PCR methods with filtering criteria of a fold change greater than 2 and a P-value less than 0.05. This analysis identified 546 mRNAs and 139 lncRNAs as deregulated, with 512 mRNAs and 118 lncRNAs upregulated, including TCONS00028768, ENST00000440778.1, and NR024075. Additionally, 34 mRNAs and specific lncRNAs (ENST00000414894.1 and ENST00000440778.1) were found to be downregulated [37]. Another study, also comparing AIS cases with healthy children, applied a P-value less than 0.05 and a fold change greater than 2 as filtering criteria, identifying upregulated microRNAs such as miR-1226-5p, miR-27a-5p, miR-223-5p, and miR-122-5p, and downregulated microRNAs like miR-671-5p and miR-1306-3p [58]. A separate analysis of patients with Friedreich's ataxia used similar methods, applying a P-value less than 0.05 and a fold change greater than 1.5. This study revealed deregulated microRNAs including miR-128-3p, miR-625-3p, miR-130b-5p, miR-151a-5p, miR-330-3p, miR-323a-3p, miR-142-3p, and miR-16-5p [60]. In addition, transcriptome sequencing and RT-PCR were employed in a study of degenerate disc tissues, using a fold change greater than 2 as the filtering criterion. This study identified 749 mRNAs, 70 circRNAs, 685 lncRNAs, and 56 miRNAs as deregulated. Among these, 194 mRNAs, 185 lncRNAs, 35 circRNAs, and 53 miRNAs were upregulated, while 555 mRNAs, 500 lncRNAs, 35 circRNAs, and 3 miRNAs were downregulated [39][47]. These studies demonstrate the significant molecular alterations observed in various conditions and highlight the importance of rigorous filtering criteria to identify key regulatory molecules (Figure-1 and -2).

Recent research has emphasized the potential of specific circulating circRNAs in serum as diagnostic biomarkers for scoliosis-related disorders. A study by García-Giménez et al. highlighted variations in circRNA abundance between patients with AIS and healthy controls [65]. Their findings demonstrated that the levels of three circRNAs—miR-122-5p, miR-27a-5p, and miR-223-5p—were significantly higher in patients with AIS compared to healthy individuals. Furthermore, these three circRNAs, along with miR-1306-3p, showed potential as biomarkers for differentiating AIS patients from normal controls [65].

Several studies have investigated the overall variations in the three main types of non-coding RNAs in patients with scoliosis. Additionally, enrichment analysis revealed that these differentially expressed RNAs are involved in key signaling pathways, such as FoxO, PI3K-Akt, mTOR, EGFR, and Wnt, among others [46].

In 2020, an extensive search was conducted across the PubMed, EMBASE, and GEO databases to identify studies comparing gene, miRNA, and lncRNA expression in patients with AIS and normal control mesenchymal stem cells (MSCs). The findings suggest that non-coding RNAs may play a role in a complex regulatory network. However, since these interaction pathways were only investigated in this study, further experimental validation is necessary to confirm their accuracy [66].

## Wnt Signaling Pathway

The Wnt signaling pathway was found to be overactive in bone biopsies from scoliotic patients, as evidenced by a significant elevation in active β-catenin levels [67]. Similar observations were made in a zebrafish scoliotic model, where β-catenin activity was associated with spinal deformity through the involvement of the enzyme tyrosine kinase 7 [15]. While activation of the Wnt/β-catenin pathway is known to increase bone mass, its overactivation in idiopathic scoliosis impairs the differentiation of osteoblasts into osteocytes and disrupts matrix mineralization [68][69]. Furthermore, Runx2, an early marker of bone formation, was found to be decreased in bone tissues from idiopathic scoliosis patients, indicating that bone formation was hindered due to the overexpression of the Wnt/β-catenin signaling pathway [70][71]. Excessive activation of the Wnt/β-catenin signaling pathway may play a role in the progression of scoliosis deformity by impairing the normal function of muscles, intervertebral discs, and the vertebral growth plate [72][73][74]. A possible explanation for the asymmetrical muscle contraction observed between the convex and concave sides of a scoliotic curve involves cadmodulin and its interaction with the Wnt/β-catenin signaling pathway and sclerostin expression. In patients with idiopathic scoliosis, cadmodulin levels were found to be higher on the convex side and lower on the concave side of the paraspinal muscles [75]. Calmodulin, when bound to calcium, activates myosin light chain, thereby playing a key role in the regulation of smooth muscle contraction [76]. Downregulation of cadmodulin was shown to influence calcitonin levels, which subsequently affects blood calcium levels and the activation of G proteins [71][77]. Downregulation of calcitonin results in a decrease in sclerostin expression, which subsequently activates the Wnt/β-catenin signaling pathway, thereby promoting the osteoblastic differentiation of bone marrow stem cells [78][79]. Similarly, G proteins activate the Wnt/β-catenin signaling pathway and show increased expression in the vertebral bodies on the convex side of the scoliotic spine [71][80][81].

These findings reveal that the Wnt/β-catenin signaling pathway plays a double-edged role in bone biology. Under normal conditions, this pathway helps regulate bone mass and supports the development of healthy bone cells. However, in scoliosis, the same pathway becomes overactive and starts to interfere with normal bone remodeling and coordination between the spine, muscles, and growth plates. This overactivity may actually make the spinal curvature worse over time. Therefore, it is important to better understand when and how this pathway switches from being helpful to becoming harmful. For example, this may depend on where in the body it is active, how it interacts with proteins like sclerostin and calmodulin, or how it responds to hormones like calcitonin. Clarifying these details could help explain why Wnt signaling supports bone health in some cases but contributes to spinal deformities in others [82][83][84]. While the Wnt signaling pathway has been widely studied for its role in skeletal development and spinal morphogenesis, recent studies have emphasized the crosstalk between Wnt signaling and inflammatory processes. In particular, dysregulation of Wnt activity can influence, and be influenced by, pro-inflammatory cytokines such as TNF-α, IL-6, and IL-1β, suggesting a bidirectional interaction between bone remodeling pathways and immune responses. This molecular interplay sets the stage for exploring how inflammatory cytokines may directly or indirectly contribute to the onset and progression of spinal curvature in scoliosis [85][86].

## Inflammatory Cytokines and Scoliosis

Cytokines are a group of small proteins released by immune cells that play a pivotal role in intercellular signaling, regulation of inflammatory responses, and control of cell growth. In the pathophysiology of scoliosis, inflammation is regarded as a crucial factor influencing disease progression. Numerous studies have highlighted that alterations in the levels of specific cytokines are strongly associated with the onset, progression, and severity of scoliosis. For instance, variations in the expression of pro-inflammatory cytokines such as tumor necrosis factor-alpha (TNF-α), interleukin-6 (IL-6), and interleukin-17 (IL-17) have been observed in individuals with scoliosis, potentially contributing to abnormal spinal curvature and impaired bone remodeling [87][88][89][90]. Furthermore, alterations in the levels of anti-inflammatory cytokines in scoliosis patients point to a complex network of inflammation regulation throughout the progression of the disease. This network appears to involve not only an imbalance between pro-inflammatory and anti-inflammatory factors but may also be influenced by the patients' genetic predispositions, immune status, and various environmental factors [91][92][93]. Therefore, gaining a deeper understanding of the role of cytokines in scoliosis is crucial for uncovering the disease's pathogenesis and identifying potential therapeutic targets, which could have substantial theoretical and practical implications.

However, it remains unclear whether inflammatory shifts are a cause of spinal deformity or a consequence of biomechanical stress due to curvature. Some studies suggest that chronic spinal loading and vertebral asymmetry in scoliosis may stimulate localized inflammation, particularly in paraspinal tissues, thereby elevating pro-inflammatory cytokine expression [94][95][96]. Conversely, other evidence proposes that a pre-existing immune dysregulation or systemic inflammatory tendency—perhaps genetically predisposed—may initiate or accelerate spinal curvature via its effects on bone metabolism and matrix remodeling [49]. A deeper exploration into this bidirectional relationship could help clarify whether cytokine alterations are primary drivers or secondary amplifiers of scoliosis, thereby informing targeted anti-inflammatory strategies for early intervention.

## Epigenetics

Epigenetics refers to modifications in gene expression that do not involve changes to the underlying DNA sequence and can be passed on through both mitotic and meiotic cell divisions [97]. The primary epigenetic mechanisms include DNA methylation, histone modification, non-coding RNA involvement, and chromatin remodeling, all of which play a crucial role in regulating gene expression and influencing cellular function and development [97]. Epigenetic modifications impact gene expression at various stages, including replication, transcription, and translation. These alterations are associated with the development of numerous conditions, such as cancer, neurodegenerative diseases, and autoimmune disorders [98][99]. Recent studies on adolescent idiopathic scoliosis (AIS) suggest that genetic variations contribute to only about 2%-3% of the causative factors, implying that other factors, such as epigenetic modifications, may play a more significant role in the development of scoliosis [100].

### Chromatin Remodeling

Chromosomes are composed of nucleosomal units, in which DNA is coiled around histones. This structure facilitates the highly condensed and organized arrangement of the genome within the cell nucleus [101]. Chromatin remodeling complexes (remodelers) alter the structure of nucleosomes by utilizing the energy from ATP hydrolysis. These complexes are crucial for processes such as transcription, DNA replication, and repair. Moreover, the ongoing remodeling of chromatin is largely dependent on these complexes, which facilitate the dynamic regulation of chromatin structure and function [101]. This mechanism safeguards genes by keeping essential gene regions protected when they are not actively in use, while also controlling the precise duration of gene exposure during replication and transcription. Such regulation helps preserve genomic stability and prevents interference with vital gene functions [101]. Chromatin remodeling is a vital epigenetic process that controls gene expression and has been linked to a range of disorders, such as cerebro-oculo-facial-skeletal syndrome and Williams-Beuren syndrome. It is also implicated in tumorigenesis and the invasion of cancer cells [102][103].

### DNA Methylation

DNA methylation in the human genome predominantly occurs at the cytosine residue of the CpG dinucleotide, which is commonly located in the promoter regions of genes, serving as a key marker for the initiation of gene transcription [97]. Under normal conditions, DNA methylation maintains a dynamic equilibrium, where the silencing of specific genes, driven by physiological requirements, regulates gene expression and supports homeostasis [104][105]. In pathological conditions, abnormal DNA methylation can interfere with gene expression, resulting in the dysregulated expression of crucial downstream products. This disruption can initiate irregular cell proliferation or apoptosis, thereby contributing to the development of scoliosis.

### Differential Methylation of Key Loci

Impairments in key enzymes or fundamental elements necessary for proper spinal growth and development may underlie the pathogenesis of spinal dysplasia, potentially predisposing individuals to the initiation or progression of scoliosis. Cartilage oligomeric matrix protein (COMP), an integral constituent of the extracellular matrix, plays a pivotal role in cartilage formation. Multiple investigations have demonstrated a marked decrease in COMP secretion among patients with adolescent idiopathic scoliosis (AIS) relative to unaffected individuals [106]. The investigation assessed both the methylation profiles and gene expression levels of COMP across five CpG sites within the COMP locus in individuals diagnosed with adolescent idiopathic scoliosis (AIS) and in healthy control subjects [107].

Shi et al. (2018) reported that the promoter region of the PITX1 gene exhibited hypermethylation in individuals with AIS, which was associated with a marked downregulation of its downstream gene products relative to healthy controls [108]. Methylation at six CpG sites within the promoter region of the PITX1 gene was found to be positively correlated with scoliosis severity. PITX1, a transcription factor belonging to the RIEG/PITX family, plays a crucial role in the basal transcriptional regulation of prolactin as well as in hormonally driven modifications of prolactin activity. Notably, dysregulated expression of PITX1 has been implicated in a range of skeletal pathologies [109][110][111]. In patients with congenital scoliosis (CS), elevated methylation levels in the KAT6B gene promoter were observed, along with a significant reduction in KAT6B expression [112].

Correlation analysis showed a positive association between elevated KAT6B methylation and an increased Cobb angle in patients with CS. Further research has confirmed that the KAT6B gene encodes a component of the histone acetyltransferase and MOZ/MORF protein complexes [113]. The MOZ/MORF protein complex is crucial for the early metabolism of skeletal and neuronal cells. As a result, abnormal DNA methylation at the KAT6B gene locus is thought to play a role in the pathogenesis of CS [114]. Collectively, these findings suggest that abnormal methylation of genes critical for bone formation and development plays a pivotal role in the onset and progression of scoliosis.

Aberrant DNA methylation at specific genomic loci holds promise as a predictive biomarker for scoliosis progression. In a 2018 study, Meng et al. reported that methylation levels at the cg01374129 locus were significantly reduced in patients with progressive AIS compared to those with non-progressive forms of the condition [115]. Regression analysis revealed that hypomethylation at the cg01374129 locus could serve as an independent prognostic marker for scoliosis progression. Specifically, the methylation level at this locus showed a sensitivity of 76.4% and a specificity of 85.6% in distinguishing between progressive and non-progressive scoliosis cases. These findings suggest that DNA methylation status holds considerable promise as a novel prognostic biomarker. The cg01374129 locus is situated in proximity to the gene encoding hyaluronan synthase 2 (HAS2), a key enzyme involved in the formation of intervertebral discs and vertebral bodies during development, as demonstrated in rat models. Aberrant methylation at this site may impair the normal development of these spinal components, thereby contributing to the progression of scoliosis [116].

The progression of scoliosis may also be modulated by key components of major signaling pathways. It has been observed that the promoter region of the PCDH10 gene is hypermethylated in individuals with AIS, leading to reduced expression of PCDH10 compared to healthy controls. Furthermore, higher methylation levels of PCDH10 have been positively associated with increased Cobb angle measurements, indicating a link between epigenetic regulation and the severity of spinal curvature [117].

PCDH10 is a downstream target of p53, a pivotal regulator of cell migration; however, it does not appear to play a direct role in cartilage development [118].

Alternatively, some studies have focused on DNA methylation changes occurring specifically within the skeletal muscle tissue surrounding the spine in individuals with scoliosis. For instance, a 2020 investigation analyzed DNA methylation patterns in deep paravertebral muscle samples obtained from both the convex and concave sides in patients with AIS [119].

Methylation of the estrogen receptor 2 (ESR2) promoter was notably elevated on the concave side of the paravertebral muscles in patients with AIS when compared to the convex side. Correlation analysis revealed a strong association between variations in ESR2 promoter methylation and the development of AIS, although no direct link was found with the severity of the curvature. This study is the first to explore the potential role of local tissue DNA methylation in scoliosis pathogenesis. Additionally, Janusz et al. investigated the deep paravertebral and superficial dorsal muscles in idiopathic scoliosis patients, focusing on the regulation of differentially methylated regions (T-DMRs) in the estrogen receptor 1 (ESR1) gene [120].

Functional consequences of promoter hypermethylation may further elucidate the mechanistic basis of scoliosis development. For instance, reduced expression of PITX1 or KAT6B due to hypermethylation may impair osteoblast-osteoclast balance by downregulating transcription factors and histone acetyltransferases essential for skeletal homeostasis, potentially leading to bone modeling defects [108][121]. Likewise, HAS2 downregulation due to altered methylation at cg01374129 could disturb intervertebral disc (IVD) homeostasis, as hyaluronan is a key component for maintaining disc hydration and viscoelasticity [122]. These epigenetic disruptions could translate into vertebral instability and progressive curvature, linking molecular alterations to biomechanical outcomes in scoliosis.

### Methylation Level Differences and Pathway Regulation

Differentially methylated regions (DMRs) at various loci frequently display variations between individuals with scoliosis and healthy controls. As a result, research has expanded beyond focusing solely on abnormal methylation at specific loci to explore the broader implications of widespread methylation alterations across the genome. Studying DMRs in monozygotic (MZ) twins—who share identical genetics but may present distinct phenotypes—provides a unique opportunity to gain deeper insights into the link between abnormal DNA methylation and the development and progression of scoliosis [123]. In 2019, a study utilizing a pair of MZ twins with AIS aimed to identify their DMRs. The researchers subsequently validated the role of these DMRs in a larger cohort of 20 AIS patients and healthy controls [124].

In this study, 313 hypermethylated and 397 hypomethylated DMRs were identified. The regulation of gene expression associated with these DMRs is mainly mediated through the MAPK/PI3K-Akt signaling pathway. Previous research has shown that the MAPK/PI3K-Akt pathway plays a crucial role in osteoblast differentiation and bone formation [125][126]. Additionally, it was reported that the MAPK pathway, along with other signaling pathways, is involved in the pathogenesis of CS in affected patients [127]. These DMRs regulate the downstream expression of proteins predominantly involved in the MAPK and calmodulin pathways, both of which are essential for cytogenesis. Moreover, the calmodulin pathway directly influences osteogenesis and plays a significant role in the development of vertebral bodies in patients with scoliosis. In a study, eight pairs of MZ twins with scoliosis were enrolled to further investigate the involvement of these pathways [128].

### Histone Modification

Histone modifications, including methylation and acetylation, are epigenetic alterations that affect transcriptional activity by modifying the structure of chromatin, which in turn regulates gene expression [97]. A genotyping study was conducted on 500 patients with AIS and 494 age-matched controls using PCR-based Invader analysis [129]. The results revealed a strong association between the rs12459350 variant, which regulates histone lysine 79 (H3K79) methylation, and susceptibility to AIS. However, the study did not explore the specific mechanisms driving this association.

In 2019, histological and genetic testing was conducted on articular cartilage from 11 patients with idiopathic scoliosis (IS), with the results compared to those of 10 matched controls. The findings suggest that histone methylation may play a role in abnormal chondrocyte proliferation through the miR-15a/Bcl2 signaling axis, thereby disrupting spinal growth and contributing to the development of scoliosis [61].

## Historical Background of Imaging in Spinal Curve Assessment

Throughout the years, much effort has been made to find an imaging technique with more advantages and fewer limitations. Wilhelm Conrad Roentgen primarily introduced the use of X-ray in assessing bony structures in 1895 [130]. Subsequently, the efforts of Dr. Godfrey Hounsfield led to the advent of computed tomography (CT) in 1973, an imaging technique widely used in different medical conditions, including abnormal spinal curvature [131]. Magnetic resonance imaging (MRI) was the next advancement in medical imaging, yielded by Paul Lauterbur in the early 1970s, which facilitated a more detailed inspection of different body parts, including the spine [132].

On the other hand, given that the studies showed inter- and intra-operator differences in the measurement of specific values used for diagnosing spinal curvature (e.g., the Cobb angle), the more recent efforts are aimed at computer-aided methods, which reduce subjective errors. Although some non-imaging methods exist, traditional imaging remains essential, with a demand for fully automated Cobb angle measurement software to enhance diagnostic accuracy and streamline scoliosis management [130].

While molecular alterations shed light on the pathogenesis of scoliosis at the cellular and genetic levels, these insights must be translated into clinical practice through thorough physical examination. The following section delves into clinical techniques essential for identifying external manifestations of underlying molecular dysfunctions.

## Detailed Review of Current Measurement Methods

Thanks to recent advances in imaging and measurement, assessing and monitoring spinal curvature has become much more precise and accessible. These techniques are essential tools for spotting conditions like scoliosis and help physicians monitor any changes over time. This paper looks at some of the most commonly used methods, exploring the unique features of each method. Table-1 offers a quick comparison, laying out the pros and cons of each method to show how they work in real-world settings.

## Clinical Examination Techniques

**Table:** Table1. Summary of Imaging Techniques for Spinal Curvature Assessment

**Technique**	**Key Metrics/Parameters**	**Strengths**	**Limitations**
X-ray	Cobb Angle, Ferguson Angle	Widely available, low cost, rapid imaging	Radiation exposure, limited to static images
CT (Computed Tomography)	3D reconstruction, axial/sagittal views	Detailed anatomical visualization	High radiation, requires a supine position
MRI	3D spinal structure, soft tissue detail	No radiation, excellent soft tissue contrast	High cost, limited availability, lengthy exam time
EOS Imaging	3D surface reconstruction, standing views	Ultra-low radiation, precise 3D measurements, weight-bearing	Expensive, limited accessibility
Surface Topography	Spinal contour, symmetry	Radiation-free, provides 3D/4D representations	Less reliable for deeper structures, limited accuracy
Ultrasound (2D/3D)	Cobb Angle, vertebral rotation	No radiation, portable, suitable for mild cases	Limited in obesity, time-consuming for 3D imaging
Motion Analysis	Gait patterns, compensatory mechanisms	In-depth dynamic assessment of movement	Requires specialized equipment, inconsistent results

Ref.s: [133-143]

The physical examination of the scoliosis patient should start with the inspection of the stature and skin before evaluating the contour of the back. Height measurement is crucial for assessing skeletal growth and the potential advancement of scoliotic curvature [144]. Moreover, particular tests will be examined in the subsequent paragraphs.

### 1. Palpation Techniques

To assess a patient suspected of scoliosis, it is essential to check for any unevenness in the shoulders and hips carefully. Significant differences in leg length, which can be checked by feeling the iliac crests or observing the alignment of the dimples at the back (formed by the posterior-superior iliac spines), may cause the spine to tilt from the pelvis, resulting in curvature [144].

### 2. Gait and Posture Analysis

Research on gait in scoliotic patients reveals several anomalies, although the results are relatively inconsistent. Mahaudens et al. documented reduced step length and restricted range of motion in the pelvis, hip, shoulder (frontal plane), and knee (sagittal plane) in scoliotic patients [145]. Chen et al. discovered that the gait patterns of scoliotic patients were analogous to those of healthy persons [146]. Additional research indicates that individuals with scoliosis may exhibit diminished cadence, restricted pelvic movement in the transverse plane, and either normal or reduced step length [147].

### 3. Physical Function Tests

Functional mobility tests (FMTs) have been validated for evaluating physical performance, trunk and lower limb muscle integrity, and body balance across several conditions, including lumbar stenosis [148]. A study by Lee et al. showed that mobility function was considerably more compromised in patients with adult spinal deformity compared to those with lumbar spinal stenosis [149]. Various studies have employed distinct FMTs for this objective: the Alternate Step Test, the Six-Meter Walk Test [150], the Sit-to-Stand Test, and the Timed Up and Go Test [150][151][152].

### 3.1. Leg Length Discrepancy Assessment

Leg length discrepancy (LLD) is common, impacting 2% to 24% of the general population and 7% to 30% of individuals with low back pain, and is associated with the development of scoliosis [153]. Measurement approaches for LLD are classified into two primary categories: direct and indirect. Direct techniques, such as the supine tape method, assess the anatomical length of each leg separately to determine the discrepancy. Indirect approaches, such as the standing lift technique, assess the discrepancy without individually measuring each leg. Furthermore, techniques may be categorized as weight-bearing (standing) or non-weight-bearing (supine/prone) [154][155]. Weight-bearing methods consider the influence of gravity on compressible tissues, whereas non-weight-bearing approaches may more accurately evaluate "true" leg length discrepancy, especially in the presence of angular deformities [153].

### 3.2. Adam's Forward Bend Test

The Adam's forward bend test, which necessitates no specialized equipment, assists in detecting scoliosis by exposing a "rib hump"—an asymmetrical back shape that signifies a curvature beyond 10 degrees and necessitates radiographic assessment [156]. The test necessitates that the subject stands and bends forward while maintaining straight knees, with arms hanging and feet and palms together. The examiner utilizes a scoliometer to assess the angle of trunk rotation (ATR). The level of ATR typically serves as a criterion for referral or subsequent imaging [157].

### 3.3. Scoliometer

The assessment of thoracic rotation or rib hump angle is a conventional method for assessing scoliosis progression in spinal clinics and school screening initiatives globally [158]. The Scoliometer, an inclinometer developed by Bunnell in 1984, minimizes the necessity for repeated radiographs by offering a dependable, non-invasive evaluation [159]. The Scoliometer is an essential instrument for monitoring scoliosis when utilized in conjunction with Cobb angle measurements. Despite the Scoliometer's association with inter- and intra-observer variability, Bonagamba et al. demonstrated optimal reproducibility by mitigating previous sources of variability, including patient placement, vertebral level palpation, and patient tiredness from repeated readings over time [160].

### 3.4. Plumb Line Assessment

A plumb line is a device commonly used to assess patients with pathological spine curvature. Plumb line distances (PDs), as delineated by Stagnara in 1988, are widely recognized and disseminated. Their interrater reliability is commendable, exhibiting a moderate correlation in identifying thoracic spine malformations, demonstrating substantial reliability and validity. Although PDs are a quantifiable method, they delineate the sagittal profile [161]. The reliability and validity of this technique, however, remain unverified and unstandardized. The plumb line approach is simple to employ; nonetheless, it is susceptible to several inaccuracies, including slight deviations, movement mistakes, and postural sway, necessitating cautious application [162].

Although physical examination provides the first clues to spinal deformities, imaging remains indispensable for definitive diagnosis and progression monitoring. The subsequent section reviews conventional and advanced imaging approaches that enhance the clinical understanding of spinal curvature abnormalities.

## Imaging Techniques

### 1. Radiographic Techniques

X-ray imaging is the gold standard for diagnosing idiopathic scoliosis due to its widespread availability, cost-effectiveness, and rapid results compared to other modalities [163]; however, children are not subjected to it for screening purposes due to radiation risks [164].

### 1.1. Cobb Angle Measurement

The Cobb angle remains the primary measure for determining how severe a spinal deformity is, particularly in cases like adolescent idiopathic scoliosis (AIS). This metric is generally used for examining the spine in the coronal and sagittal views [165]. In the standard approach, the upper and lower end vertebrae are identified on anteroposterior X-ray images of the whole spine. Afterward, vertical lines are drawn along the endplate lines of these vertebrae, and the angle created between these two vertical lines is known as the Cobb angle [166].

Limitations constrain this method; the reference vertebrae appear to differ across research, potentially resulting in varying measurements and, complicating comparisons and the creation of normative values. Arm location constitutes an additional inconsistency in the radiologic evaluation that may hinder the assessment [167].

Given these limitations, future directions should explore the integration of molecular profiling—including non-coding RNAs, methylation markers, and cytokine signatures—with imaging data to enhance diagnostic precision. Such an approach may enable the development of biomarker-imaging correlation models capable of predicting scoliosis onset and progression beyond static anatomical measurements like the Cobb angle. This convergence could open avenues for more dynamic, individualized, and mechanistic assessment strategies in clinical practice.

### 1.2. Ferguson Method and EOS Imaging

The Ferguson angle offers an alternative way to gauge the severity of coronal spine deformities [168]. It involves identifying the two terminal vertebrae at the curve ends based on Cobb angle measurements and locating the apex vertebra. Traditionally, the apical vertebra was viewed as the one with the most rotation and distortion yet with minimal tilt. The current standard, however, defines it as the vertebra with the greatest lateral shift from the central sacral vertical line (CSVL), a vertical line passing through the center of the first sacral segment. The angle known as the Ferguson angle is then formed by drawing lines between the midpoints of the terminal and apical vertebrae [169].

### 1.3. Risser Sign

The Risser sign is not primarily used to diagnose scoliosis but to understand its progression. This metric evaluates the ossification level of the iliac apophysis to give a semi-quantitative view of a patient's skeletal maturity [170]. The iliac apophyses' ossification usually happens closely with the vertebral ring apophyses, allowing for an estimation of the spine's remaining growth potential. In idiopathic scoliosis, progression often peaks during adolescence, but the prognosis improves with advanced skeletal maturity, as shown by higher Risser stages. Typically, ossification of the iliac apophysis can be seen on radiographs in adolescents aged 12 to 15 [169].

### 1.4. Nash-moe Method of Vertebral Rotation

The Nash and Moe method is used to assess the degree of rotation in the apical vertebra, which is the vertebra with the highest rotation and lateral shift within a curve [171]. This rotation causes both pedicles of the apical vertebra to move toward the curve's concave side. The Nash and Moe system divides the vertebral body into six sections and rates pedicle rotation on a five-point scale [169].

### 1.5. Whole-spine Standing Radiographs (EOS Imaging)

The EOS X-ray system provides biplanar images of the entire body in a standing, weight-bearing position with minimal radiation exposure. By capturing both front and side views, the EOS system enables a 3D reconstruction of the skeleton [172]. This approach offers highly accurate measurements of skeletal structures, including limb lengths, angles, and spinal curvature (such as kyphosis, lordosis, and scoliosis), presented in a true-to-size 1:1 scale [173].

### 1.6. Limitations of X-ray

While X-rays are effective for measuring spinal curvature, they fall short in assessing the cosmetic impact of deformity in patients with AIS. During adolescence, many individuals are more concerned about correcting the visual appearance of their back rather than the degree of spinal curve [174].

### 1.7. Coronal Trunk Balance

The balance of the spinal column, particularly in the frontal plane, can be indicated by the lateral trunk deviation. A vertical line is dropped from the center of the C7 vertebral body to the baseline on a full-spine X-ray. The distance between this line and the CSVL—a vertical line through the center of the first sacral segment—represents the coronal trunk balance [175]. When the plumb line shifts left, the value is negative; when it moves right, the value is positive [169].

### 2. Surface Topography Techniques

Developing a system for identifying and monitoring scoliosis is crucial to minimize exposure to ionizing radiation, hence decreasing the risk of malignant diseases in patients. Surface topography (ST) is an imaging technique that requires no supplementary apparatus or equipment, rendering it an appropriate option for various clinical settings. These techniques produce a 3D/4D representation of patients' spines utilizing diverse models and protocols, enabling the quantification of the cosmetic deformity associated with AIS while avoiding exposure to ionizing radiation [174].

### 2.1 Moiré Topography

The Moiré technique, an early method of surface topography, employs overlapping patterned grids projected onto the rear surface. This projection delineates contour variations, facilitating the evaluation of spinal curvature [176]. The Moiré approach, albeit valued for its simplicity and cost-effectiveness, is constrained by inconsistent accuracy, which hinders its exclusive application in clinical environments. It is recommended as an adjunctive approach to radiography to minimize radiation exposure, which is particularly advantageous for the longitudinal scoliosis assessment [174].

### 2.2 Rasterstereography

Rasterstereography, subsequently developed, enhanced surface measuring by employing a slide projector to project gridlines onto the posterior surface. The distortions in these lines, captured by a camera, generate a three-dimensional reconstruction of the surface of the back. Devices such as ISIS and ISIS2 enhanced rasterstereography, optimizing acquisition duration and minimizing the impact of motion artifacts [174].

Rasterstereography is primarily characterized by two measurement methods: (1) the first employs the analysis of light projected onto the subject's skin, which is dependable and constitutes the most prevalent application of rasterstereography; (2) the second utilizes an infrared and time-of-flight 3D RGB camera, which also appears to be reliable [177]. However, the method's constraints, including vulnerability to postural alterations, have hindered its practical implementation [174].

### 2.3 Formetric 3D/4D

The Formetric 3D system, an advancement of rasterstereography, initially faced challenges with dependability owing to postural wobble. Formetric 4D mitigated this issue by averaging several images to diminish motion artifacts. This approach has shown a robust association with radiographic Cobb angle measures, affirming its utility in scoliosis monitoring rather than initial diagnosis. The Formetric systems demonstrate commendable test-retest dependability; nonetheless, they are prohibitively expensive for regular monitoring [174].

While radiographic methods have long been the cornerstone of scoliosis evaluation, concerns about radiation exposure—especially in pediatric patients—have prompted the development of alternative imaging strategies. These radiation-free modalities are discussed in the following section.

### 3. Ultrasound Techniques

Ultrasound (US) imaging has gained attention in recent years due to its non-radiative nature, ease of use, and affordability, making it a valuable tool for scoliosis research. Numerous researchers have explored and developed US imaging, recognizing its potential as a leading methodology in this field [178].

### 3.1. 2D Ultrasound

Ultrasound provides a clear view of the spine's posterior surface and is generally easier to access than MRI or radiography. Portable ultrasound devices could enable spine monitoring in areas without fixed medical imaging facilities. Research has revealed a consistent relationship between the Cobb angle measured on X-rays and vertebral rotation identified by ultrasound at the apex vertebra in untreated scoliosis patients [179]. Additionally, by integrating tracking capabilities into the ultrasound transducer, clinicians can now reconstruct 3D volumes from 2D ultrasound images, opening new possibilities for spinal diagnostic assessments [180].

### 3.2. 3D Ultrasound

Developed by Suzuki et al., 3D spinal ultrasonography has demonstrated efficacy for AIS [181]. Significantly, Chen et al. [182] validated the "center-of-lamina" methodology, demonstrating that it yields curve magnitude and vertebral rotation data analogous to traditional radiography.

Grounded in the premise that the laminae and spinous processes function as dependable reference points, it offers a method for evaluating three-dimensional spinal abnormalities by analyzing vertebral rotation in relation to the orientation of the laminae and the ultrasound sensor [179]. Li et al. (2012) conducted a study on the efficacy of orthotic treatment for patients with AIS utilizing 3D ultrasonography to assess the spinous process angle, aiming to improve orthotic treatment outcomes. The findings indicated that the ultrasound-assisted fitting technique for spinal orthoses was effective and advantageous for 62% of the patients [183].

Ultrasonography is a readily accessible method that offers the benefits of being radiation-free and cost-effective. The limitations include restricted identification of lower-degree curves and an increased likelihood of human mistakes. Nonetheless, it can facilitate the secure assessment of curve progression over time without necessitating repeated radiography observations at short intervals [184].

### 3.3. Ultrasound-based Scolioscan

Scolioscan utilizes ultrasound imaging to generate three-dimensional spine models, providing a dependable radiation-free option. This approach demonstrates a strong association with radiographic Cobb angles, particularly in mild scoliosis cases. Nonetheless, its extended acquisition duration and potential difficulties in imaging obese people are disadvantages. The technique is efficient for static measurements but is inadequate for dynamic activities, akin to Rasterstereography [174].

### 3.4. Elastography (Ultrasound-based)

Various non-invasive methods now exist to measure the elasticity of tissues, helping to understand their mechanical properties. These elasticity imaging techniques gather data on tissue flexibility and can be applied to deeper organs, opening up new possibilities for screening and diagnosis [185]. In the 1970s and 1980s, early approaches used static loading and external vibrations to apply stress to tissues, followed by modified color Doppler to track tissue movement and measure stiffness [186][187]. By the late 1990s, a quasi-static method was developed to assess tissue elasticity remotely through physical compression or natural body pulsations, a technique now known as strain elastography [188]. Later on, dynamic shear wave elastography emerged, allowing the measurement of shear wave speed (SWS), which correlates directly with the tissue's elastic properties, unlike strain elastography [189]. Shear wave elastography uses focused acoustic radiation to generate shear waves within the tissue, measuring the wave speed to assess local stiffness [188][190].

### 3.5. Automatic Spine Ultrasound Segmentation

Automated Spine Segmentation and Measurement is a novel, AI-based method that utilizes monitored ultrasonography and convolutional neural networks (CNNs) to evaluate spinal curvature. This technology utilizes CNNs to autonomously detect and segment the spine from ultrasound pictures, thereby generating a 3D spinal model for precise scoliosis assessment. This automated procedure requires under one minute and attains a maximum error margin of approximately 2.2° compared to conventional X-rays [191].

### 4. Alternative Imaging Methods

### 4.1 Photogrammetry

Photogrammetry is a dependable method for acquiring information about an object and its surroundings through the measurement and analysis of photographic images, facilitating the quantification of human body measurements [167]. It facilitates precise quantitative assessment by documenting subtle alterations in postural alignment [192]. This method may be deemed superior to alternative non-invasive techniques due to its low cost, ease of transport and photo-interpretation, and capacity to measure minor postural alterations while tracking the progression, stabilization, or reduction of postural asymmetries in adults over time [193]. Although it is a straightforward procedure employed extensively, it has certain disadvantages, namely that it is time-consuming and does not yield quick results. Furthermore, being a two-dimensional technique, it cannot evaluate rotational differences among vertebrae [162]. Moreover, research has indicated elevated intra- and inter-rater dependability for the photogrammetric approach [194][195].

### 4.2 Spinal Mouse

The skin-surface mouse is a viable and trustworthy instrument for spinal evaluation, particularly for kyphotic posture. It can be maneuvered along the spinal profile to measure vertebral shape and angulation. The Spinal Mouse is cost-effective; however, its price range remains inaccessible to some; it offers great precision and robust software analysis, while it is exclusively concentrated on the spine [177].

### 4.3. Motion Analysis Systems

Progress in dynamic motion analysis offers a more thorough evaluation of gait and balance. Skalli and associates were among the first to employ motion analysis to detect dynamic compensations in scoliosis patients, highlighting the pelvis's significance in postural control before and during surgical intervention [196]. Patel et al. expanded this research by assessing pelvic incidence as a predictor of sagittal alignment and hip dynamics, noting that elevated pelvic incidence was associated with an augmented hip range of motion. Their findings indicate that pelvic morphology affects gait patterns, highlighting the necessity for patient-specific motion analysis in conjunction with conventional imaging to enhance personalized surgery planning [197].

### 4.4. CT scan

CT has restricted utility in scoliosis diagnosis due to its carcinogenic potential and the requirement for the supine position during imaging. The supine position alters the existing three-dimensional spinal malformation. A 3D representation of the standing position provides precise findings for scoliosis diagnosis [184]. In accordance with standard practices in most institutions, a prone position during CT scanning will be employed to replicate the surgical position closely. EOS serves as an option to address these restrictions [198].

### 4.5. MRI

Non-radiative options, such as sonographic analysis, can only partially evaluate the situation [199]. MRI has been recognized as a comparable alternative for evaluating Cobb angle [200][201]. Regrettably, MRI is less accessible, more costly, and necessitates an examination duration of 20-60 minutes, during which the patient must avoid excessive movement [202]. This scenario may pose difficulties for younger children; however, a recent study indicates that MRI can still yield pertinent information [203]. A revolutionary, rapid, low-angle shot MRI technology (FLASH 2.0) now offers a radiation-free, ultra-fast alternative to radiography that is suitable for daily usage and unaffected by mobility [204][205].

Additionally, a recent study showed that real-time MRI offers diagnostic efficacy comparable to traditional radiography in assessing idiopathic scoliosis, while eliminating the need for ionizing radiation. The duration of an MRI examination is slightly shorter than that of traditional radiography. Therefore, spinal real-time MRI assessment serves as an excellent and efficient alternative to conventional radiography [206].

Nonetheless, the application of MRI is constrained. If screws, hooks, or rods are implanted in the subject's body for spinal correction, an MRI cannot be performed [184]. The routine use of MRI in idiopathic scoliosis remains a topic of debate, as the indications for its application vary across studies. However, the established criteria for the routine use of MRI can be summarized as follows: presence of pain (back, neck, radicular, headache), neurological findings (such as clonus, abnormal abdominal reflexes, weakness, urinary dysfunction, hyperreflexia, asymmetric deep tendon reflexes, paresthesia, diminished rectal tone, cavus foot deformity, skin lesions), atypical curve patterns (including left thoracic, short segment, reduced rotation, absence of thoracic apical segmental lordosis, rapid progression, and a thoracic kyphosis angle >30 degrees), early-onset scoliosis, male gender, and the presence of associated organ anomalies [207][208].

Importantly, the advancement of both molecular biology and imaging has opened new frontiers for integrated diagnostics. The next section explores how these two complementary approaches can be combined to improve early detection and personalized management of scoliosis.

## Bridging Molecular and Imaging Approaches in Scoliosis Diagnosis

An integrated diagnostic approach that combines molecular insights with imaging findings holds great potential for improving the early detection and personalized management of scoliosis. Molecular alterations such as dysregulated non-coding RNAs, epigenetic modifications, and imbalances in inflammatory cytokines may contribute to pathological changes in spinal development that are subsequently detectable through imaging.

For instance, aberrant expression of miR-122-5p, miR-27a-5p, and miR-223-5p has been associated with adolescent idiopathic scoliosis and may reflect underlying structural abnormalities that can be visualized through 3D ultrasound imaging or surface topography [65][67]. Similarly, overactivation of the Wnt/β-catenin pathway, which impairs bone matrix mineralization, correlates with abnormalities detected by EOS imaging and Cobb angle progression [52][67].

By correlating molecular biomarkers with radiological features—such as curve magnitude, vertebral rotation, or paraspinal asymmetry—clinicians may be able to identify high-risk patients earlier and tailor monitoring and intervention strategies. This convergence of biological and structural data represents a critical step toward precision medicine in scoliosis care.

## Conclusion

In conclusion, integrating molecular insights with advanced imaging methodologies offers a promising avenue for the early diagnosis and personalized management of pathological spinal curvature. The evidence indicates that the dysregulation of non-coding RNAs, overactivation of the Wnt/β-catenin pathway, and imbalances in inflammatory cytokines and epigenetic modifications significantly contribute to the development and progression of spinal deformities. Concurrently, the evolution of imaging techniques—from traditional radiography to state-of-the-art 3D reconstruction and computer-assisted measurements—has markedly enhanced the precision of spinal curvature assessment.

Looking forward, proposing ncRNA-based and methylation-based biomarkers holds significant potential for the early prediction or monitoring of scoliosis progression. Moreover, bioinformatic approaches, such as integrative transcriptomic and methylation analyses, may facilitate the discovery of novel molecular subtypes of scoliosis, paving the way for stratified and more effective therapeutic strategies. Future research that further bridges these molecular and clinical domains is essential for devising targeted interventions that effectively address both the biological and structural components of spinal curvature, ultimately leading to improved patient outcomes.

## Conflict of Interest

There is no conflict of interests.
